# “I Just Wanted a Dentist in My Phone”—Designing Evidence-Based mHealth Prototype to Improve Preschool Children’s Oral and Dental Health: Multimethod Study of the Codevelopment of an App for Children’s Teeth

**DOI:** 10.2196/49561

**Published:** 2024-01-30

**Authors:** Waraf Al-yaseen, Daniela Procida Raggio, Mariana Araujo, Nicola Innes

**Affiliations:** 1 School of Dentistry Cardiff University Cardiff United Kingdom; 2 FDI World Dental Federation Geneva Swaziland

**Keywords:** oral health promotion, mobile health, mHealth, children, oral health, behavior change, coproduction, mobile phone

## Abstract

**Background:**

Dental caries in preschool children is a global health concern. With increased access to technology and the disruption of health care during the pandemic, mobile health apps have been of interest as potential vehicles for individuals’ health maintenance. However, little is known about caring for their child’s teeth and what their preferences would be regarding the content or design of an oral health app.

**Objective:**

This study aims to co-design the prototype of an app named App for Children’s Teeth with parents, providing a source of information for them about caring for their children’s teeth and promoting positive dental habits.

**Methods:**

This multimethod study conducted user involvement research with a purposive sample of parents or carers of children aged ≤6 years to (1) understand their use of the internet through the eHealth Literacy Scale and interviews, (2) determine their opinions about content related to children’s oral health, and (3) collect feedback about the app’s acceptability using the Theoretical Framework of Acceptability. There were three stages: (1) interviews with parents to understand their needs, preferences, and abilities; (2) prototype design with app developers; and (3) parent feedback interviews using the think aloud method for data collection. Data were deductively analyzed using a codebook strategy, whereas data from the think aloud sessions were analyzed inductively using reflexive thematic analysis.

**Results:**

The prototype design stage involved 10 parents who reported using the internet for health information but found it to be scattered and contradictory. Parents generally welcomed the *App for Children’s Teeth* but expressed concerns about screen time and practicality. They suggested guidance regarding oral hygiene practices, teething symptoms, and pain relief. Parents appreciated features such as clear fonts, categorization according to their child’s age, and “In a Nutshell” bullet points. Topics that resonated with parents included information about teething, finding a dentist, and breastfeeding. They believed that the app aligned with their goals and offered suggestions for future developments, such as outlining the process of finding a dentist and incorporating a forum for parents to communicate and exchange ideas.

**Conclusions:**

The coproduction design approach highlighted parents’ need for solutions such as mobile health apps to access reliable information about oral health. Parents identified key design concepts for the app, including a simple and uncluttered interface, content categorization according to their child’s age, and practical guidance supported by visual aids. Despite potential challenges related to screen time restrictions, parents provided insights into how such an app could fit seamlessly into their lives.

**Trial Registration:**

Open Science Framework; https://osf.io/uj9az

## Introduction

### Background

Dental caries is the most common chronic disease of childhood, affecting >621 million children worldwide [1]. It negatively affects the quality of life of children and their parents [2]. The condition, dental caries, is biofilm mediated, diet modulated, multifactorial, and noncommunicable. It is dynamic in nature; it can start and stop depending on external factors (such as biofilm removal), and it can progress and regress (demineralization and remineralization), again depending on external factors such as the amount and frequency of sugar in the diet [3], removal of the biofilm, and fluoride application to the teeth during toothbrushing [4]. Evidence-based guidelines recommend that parents take responsibility for their children’s daily activities, care, and food choices to prevent dental caries [5]. These guidelines emphasize managing the external factors through personal care, including toothbrushing with fluoridated toothpaste (>1000 parts per million) and sugar reduction in food choices [5,6].

In recent years, the development and growth of mobile health (mHealth) apps have provided an opportunity to improve access to health information and promote health and well-being using mobile technologies [7]. mHealth has been defined by the World Health Organization as the “use of mobile and wireless technologies to support the achievement of health objectives” [8]. Smartphones are ubiquitous, with an estimated 88% of adults owning one in the United Kingdom in 2021 [7]. Internet use exclusively through smartphones is increasing, and in 2022, overall, 21% of users accessed the internet exclusively through their phones. As such, there is potential for high-quality and engaging health information to be easily accessible and affordable through mHealth. Although there is evidence that mHealth apps can play a role in improving health outcomes, including for oral health and dental plaque control [9], the quality and accuracy of internet-based health information is variable, with potential for harm from misinformation.

To maximize the potential of mHealth apps, it is crucial to prioritize user engagement by involving target stakeholders such as patients, caregivers, and health care providers in the co-design process. Co-design fosters collaboration and leverages the valuable insights of users and designers in the development of products or services. By incorporating user feedback and preferences into the design process, mHealth apps can better meet the needs of their target audience. Theoretical Framework of Acceptability (TFA) is a valuable tool to guide the co-design process. TFA proposes that the acceptability of any intervention, such as an mHealth app, is determined by its perceived appropriateness, effectiveness, and feasibility [10]. TFA provides an evidence-based checklist to assess these individual components in an mHealth app prototype, allowing deficiencies to be addressed and ensuring that it meets the needs and preferences of the target stakeholders, and guides the next steps in the development process.

As co-design is a user-centered process, it considers the target audience’s specific characteristics. Context, education, age, and eHealth competence of the end users, among other factors, can significantly influence the design and structure of mHealth apps. The eHealth Literacy Scale (eHLS) is a tool that measures individuals’ ability to access, comprehend, and use health information to make health care judgments. By considering the eHealth literacy of the target stakeholders, an app can be designed to be more appropriate, accessible, and user-friendly, thus improving the likelihood of it being used and, ultimately, its effectiveness [11].

The development of an mHealth app for parents and caregivers of young children that addresses gaps in knowledge and presents evidence-based information in a usable format might promote healthy oral hygiene practices and prevent dental caries in children.

### Aims and Objectives

This study aimed to provide proof of concept for designing eHealth technologies in collaboration with the parents of young children, as end users, by designing a prototype of a medical health app to identify gaps in information, develop content, and collect feedback about acceptability.

The objectives were to conduct user involvement studies with parents or carers of children aged ≤6 years at the initial stage to do the following:

- Understand their use of the internet through eHLS and interview
- Determine their opinions about mHealth app content related to children’s oral health

Then, at a later stage of development of the medical health application, we aimed to do the following:

- Collect feedback about the acceptability of the medical health application according to the domains of TFA

## Methods

### Design

This was a single-site, multimethod, qualitative research study that evaluated parents or carers’ acceptability of a smartphone app prototype.

### Ethical Considerations and Data Management

A favorable ethical opinion from Cardiff School of Dentistry Research Ethical Committee (reference number 2210a) was provided by the Dental School Research Ethics Committee on September 13, 2022. The study was conducted from October 2022 to May 2023. The project ensured participant safety and privacy by conducting interviews in safe and private locations, implementing measures to keep both participants and researchers safe, and maintaining confidentiality of personal information. If sensitive information or harm was disclosed, the research team collectively decided about the further steps and documented the process. Participants seeking dental advice were directed to National Health Service (NHS) Wales. No identifiable information was used in reports or research papers. Study protocol was registered on the Open Science Framework.

All information collected from participants during the study was maintained strictly confidential, and any personal information they provided was managed according to Cardiff University requirements and following General Data Protection Regulation recommendations for data protection (2018) [12]. Records and study documents were stored in a password-protected folder within the university’s OneDrive (Microsoft Corp) cloud space and were accessible only to the research team. Paper-based consent forms were stored in a secure, locked drawer within a locked room on the university premises. Anonymized information and consent forms will be retained for 5 years before being destroyed. However, it could be retained indefinitely when it is likely to have continuing value for research purposes. None of the individuals were identifiable from the data in the reports provided to the funders and partners or in the published research papers

### Participants

#### Sample Size

A sample size was not set a priori with recruitment continuing until the research team agreed that the data set was sufficiently comprehensive and rich to address the research objectives. On the basis of similar research projects, the sample size was expected to be approximately ≤10 parents or carers [13].

#### Inclusion Criteria

Adults (aged ≥18 y) who (1) were parents or carers of children aged ≤6 years; (2) were willing to attend 2 in-person interviews; and (3) considered themselves to be fluent in English, Arabic, or Portuguese were eligible to participate.

#### Exclusion Criteria

Anyone who was working or had worked in the dental profession in any capacity was not eligible to participate.

### Procedure

#### Overview

**Figure 1:**
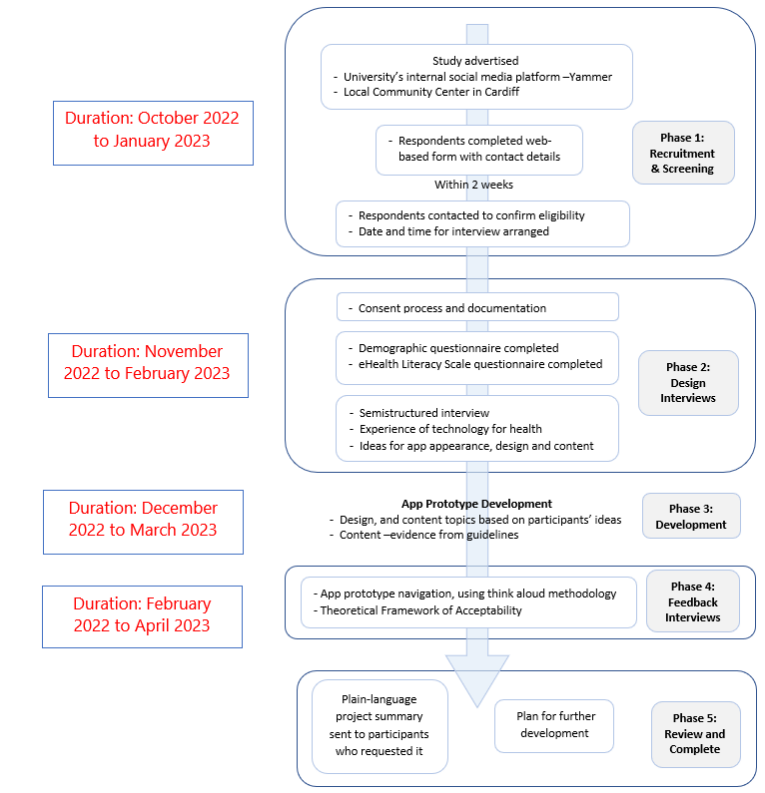
An overview of the study’s mixed methods methodology—participant journey and data collection.

An overview of the project design is shown in Figure 1. In this study, a multimethod approach was used to comprehensively investigate the research question. Multiple qualitative methods were used, consisting of structured interviews conducted during the 2 stages of data collection, complemented by think aloud methods during feedback interviews. The structured interviews were designed in accordance with TFA, enabling a systematic exploration of various acceptability dimensions. Concurrently, think aloud methods provided real-time insights into participants’ interactions with the app. This integrated approach aimed to thoroughly assess parental perceptions about app acceptability and elucidate the underlying factors. The structured interviews offered a systematic framework, whereas think aloud methods provided spontaneous insights, augmenting the depth and validity of the analysis.

The study used structured interviews as the primary data collection method. These interviews followed a standardized format, with predetermined questions aligned with the research objectives and the TFA. The structured interview approach ensured consistency across all interviews and facilitated a comprehensive exploration of acceptability dimensions. The interviews were conducted in 2 stages to capture the initial impressions and feedback after app use and were set up in one-to-one format between the participant and one of the research team members (DPR or WA). They were conducted at a mutually agreed time and in either a quiet room in the university or at a community center, depending on participants’ preferences. Both were appropriate for conducting interviews—safe, accessible, and private.

#### Phase 1: Recruitment

##### Overview

A purposive recruitment strategy was adopted, in which 2 different routes were targeted (Cardiff University employees and attendees of a community center). A web-based invitation was posted on the “Taking Part in the Research” Cardiff University Yammer and on Cardiff University’s social media pages and shared within the researchers’ local community networks (Multimedia Appendix 1). Recruitment from Grangetown Community Centre was conducted by attending public engagement events, such as community fairs and local meetings, and by directly approaching the attendees. The researchers worked with the staff at the community center to help coordinate the recruitment and data collection efforts.

##### Participant Screening, Information Sharing, and Consent Process

Figure 1 describes the process further. The web-based invitation for both groups referred interested individuals to a web-based questionnaire for further information (Multimedia Appendix 2). This also acted as a first-line screen to guide potential participants regarding whether they met the inclusion criteria and asked them to give their contact details. A member of the research team contacted each person via phone or email within 2 weeks to perform further screening to identify those meeting the inclusion criteria and to explain the study, its aim, that it involved one-to-one interviews with the researchers, and any risks and benefits anticipated by participating in the study.

If they were eligible and still interested in participating, the participant information sheet (Multimedia Appendix 3) and a consent form (Multimedia Appendix 4) were sent to the potential participants via email for them to review before meeting with the researchers during the face-to-face interview.

During the interview meetings, the interviewers discussed the study and checked the participants’ understanding, and a paper-based consent was signed if they were still agreeable to participate, before conducting the interview.

#### Phase 2: Requirement Definition and Design Interview

For the first data collection session (design interviews), participants were invited to complete a demographic questionnaire and the eHLS questionnaire. The interviewer explored with the participants the way they currently found and accessed information related to oral health, experience with using technology for health purposes, and potential ideas for the technical development of the app (Multimedia Appendix 5).

#### Phase 3: Develop a Prototype With an App Development Team

Participants’ inputs were analyzed and shared with the International Dental Federation (Fédération Dentaire Internationale) research team and the Swiss Tomato app development team to conduct brainstorming discussion meetings. The multidisciplinary team examined the results, proposed content and features for the app, and developed the blueprint for the app. The development team used the blueprint to further develop the prototype.

#### Phase 4: Feedback Interview (Assess the App’s Acceptability)

For the second data collection session (feedback interviews), the researchers maintained the same arrangements as in the first session and conducted the interview in person at a suitable and quiet location. Before the interview, a member of the research team contacted the participants to confirm the location, time, and date of the interview.

During the interview, the interviewer presented the prototype to the participant as a web page and explained how to use it (Multimedia Appendix 6). The data were collected using 2 strategies. First, “Think Aloud,” where the participants were asked to navigate the prototype while expressing their thoughts and perceptions out loud. In addition, the interviewer asked questions based on the TFA theory at this stage. These questions may have focused on the user’s attitudes and perceptions toward the prototype, such as its perceived usefulness, ease of use, and intention to use the system.

### Data Handling and Analysis

Recordings were transcribed verbatim using a Cardiff University–approved service. The research team anonymized all personal data collected from or about the participants, except for signed consent forms. Personal information in transcripts that could identify the participants was masked with pseudonyms or omitted if it did not affect the transcript’s context.

Descriptive analysis was used for the participant demographic questionnaire and eHLS data. Qualitative data were analyzed separately for each data collection session using NVivo software (Lumivero) under the Cardiff University license and then interpreted together using a complementary approach. Codebook thematic analysis was used for the design interviews and TFA data [10]. Data from think aloud transcripts were analyzed inductively using reflective analysis.

Participants’ demographic and eHLS details were presented in frequencies. Qualitative data were presented according to the topic summary, with direct quotations from participants’ accounts.

## Results

### Participants and Interviews

#### Demographics

The study included 10 parents, with 8 (80%) from Cardiff University and 2 (20%) from Grangetown Community Centre. Table 1 presents their respective demographic characteristics.

**Table 1:** Participant demographics and interview summary information (n=10).

Characteristics and groups			Participants, n (%)
**Gender**			
	Man	2 (20)	
	Woman	8 (80)	
**Age (y)**			
	25-34	2 (20)	
	35-44	8 (80)	
	≥45	0 (0)	
**Self-identified ethnicity**			
	White British	7 (70)	
	White non-British	1 (10)	
	Other	2 (20)	
**Education**			
	Attended college course	1 (10)	
	Attended university course	7 (70)	
	Other postgraduate education	2 (20)	

#### Interview Characteristics

All participants (10/10, 100%) participated in interview 1. The interviews ranged from 17 to 58 (mean 29.2, SD 11.4) minutes and lasted for a total duration of 292 minutes. However, for interview 2, a participant was unable to attend any of the proposed dates or times owing to a family illness. Therefore, 90% (9/10) of the participants was interviewed during this phase. The duration of the interviews varied, ranging from 23 to 52 (mean 31.1, SD 11.4) minutes and lasting for a total of 280 minutes.

### Phase 2: Understand Parents’ Needs and Their Thoughts About and Experience With mHealth Apps

Parents were asked to fill an eHLS survey before the first interview. The survey was completed by 90% (9/10) of the participants. Most of them (7/9, 78%) thought that the internet was useful or very useful in helping them to make health decisions, and 89% (8/9) of the parents felt that it was important or very important for them to be able to access health resources on the internet (Multimedia Appendix 7).

**Figure 2:**
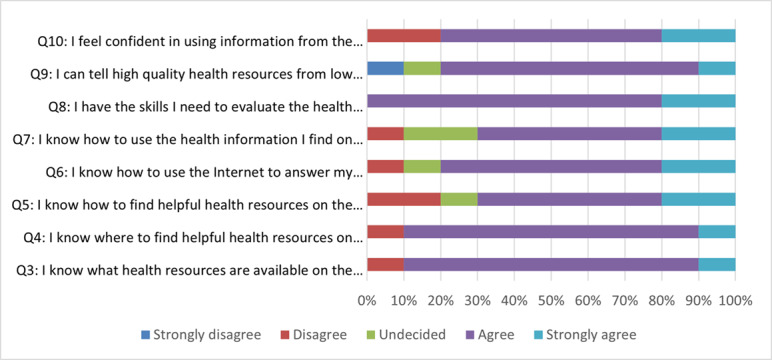
Participants’ responses to the eHealth Literary Scale questions (Qs) 3 to 10 (n=9).

The remaining 7 questions (Figure 2) showed that most parents (7/10, 70%) agreed or strongly agreed with the positive statements about their abilities related to using the internet for health purposes.

### Thematic Analysis of Interviews

#### Overview

The topic guide prompted the parents to discuss several key areas. First, they shared insights about their current sources of information, highlighting both the positive and negative aspects of the different sources they used. Second, they reflected upon their previous experiences with mHealth apps. Finally, they expressed their opinions about the development of an app to assist them in caring for their children’s teeth. During the discussions, the parents freely discussed a range of topics, which could be categorized into three main groups: (1) general comments, encompassing the perceived need for such an app and its compatibility with their lifestyles; (2) desired content and features they would like to see in the app; and (3) preferences regarding the app’s design and visual elements.

#### Current Sources of Information

Parents stated that they consulted many sources for a broad range of oral health information, with a focus on teething, oral hygiene practices, and identifying oral conditions. These searches were mainly through the internet, especially Google and government websites such as the NHS website. Although they perceived this as an easy-to-access and readily available tool to find information and give peace of mind regarding a topic, they highlighted several issues, including the often scattered and sometimes contradictory nature of the information on the internet:

...It’s so segregated, so almost every question you have is a separate search.

Parent 8

Another issue they felt was that the information they found was very general in terms of the management without appreciating the variety of normal presentations:

...The problem with looking on the Internet for a picture of a condition is you’re often shown the worst example and also, you’re not showing the full spectrum of possible images...various health conditions can present in various different ways.

Parent 4

They also felt that technical details about oral hygiene advice were often lacking:

The main things around the technicalities of it [Brushing]...Everything from selection of toothbrush, the firmness of bristles, the length of brushing and whether the age range that it’s safe for a child to brush themselves.

Parent 6

There was a perceived lack of information on what parents thought were reliable sources of information, such as NHS website, especially about common oral issues that affected them, such as teething. Web-based forums such as Mumsnet and Facebook groups such as Gentle Parents were also commonly used, providing interactive, specific tips from other parents. A participant found Facebook groups to be particularly helpful for finding green alternatives to oral products:

They are [Gentle Parents Facebook group], generally very ethical and environmentally aware and quite into natural products rather than more standardised.

Parent 9

When it comes to credibility of other parents’ advice, parents used their experience to judge:

...If it resonates with my experience, then I would probably go ahead with that.

Parent 9

#### Parent’s Previous Experience With mHealth Apps

The parents who were interviewed shared a diverse range of experiences with mHealth apps. Mental health apps, particularly the Headspace app, were the most common types of apps that parents had used. Some parents stated that they downloaded the app because their university provided free access to the “Paid” service, which they considered to be a good deal. Some parents mentioned that they still occasionally used the app as it provided easy-to-follow relaxing exercises, and they appreciated receiving regular updates and notifications. Other popular apps among the parents were the NHS COVID-19 app, which provided instant help about laboratory tests and nearest testing centers during the pandemic, and apps that focus on weight loss and tracking physical activity. None of the interviewed parents (0/10, 0%) reported using or looking for an app to help them find health information.

#### Parents’ Thoughts About the Development of an mHealth App for Oral Health

##### General Comments

Participants were positive about having access to an app to assist with children’s dental care, citing the need for a reliable and convenient source of information. They talked about potential challenges to using it. Several parents (2/10, 20%) questioned the app’s effectiveness, as people can easily search for answers to their questions on Google. They also worried about whether users would remember to use the app regularly. A possible solution was suggested by a parent: incentivizing app use by giving users “gold stars” for using it every day to encourage regular use. Another issue discussed was the challenge of integrating the app into busy morning routines. Finally, a parent noted the potential difficulty of using the app alongside other devices or apps and fitting it into their lifestyle but also tried to address these challenges with suggestions:

I still haven’t quite worked out how we could fit it in, in the morning because it’s so staggered...So, the whole idea of teeth time is this time in the morning is much more difficult to kind of work out rather than we’re all heading towards bed and therefore we’re all doing our teeth.

Parent 8

Screen time concerns were highlighted by some parents as the app is intended to be used by both parents and their child:

I think a lot of parents in this day and age struggle already with the amount of screen time. So that would be a bit of a two-edged sword for me.

Parent 5

##### Content Suggestions

Suggestions for the content of the app include accessible guidance to help identify potential oral health problems and about how to manage them. They particularly mentioned teething, including timings, managing symptoms, providing relief, and understanding when things were normal or not in development:

I just wanted a dentist in my phone...I can check whether I need to go and see the doctor, or this is something I shouldn’t be worried about.

Parent 3

They also wanted guidance and more precise details about how to perform oral hygiene practices, such as when to start brushing their child’s teeth, introducing flossing, how to handle brushing when the child is using inhalers, and importance of supervised brushing for proper cleaning:

Stuff about how you do it [brushing]? at what age do you let them have more control over it? Till when am I still doing it? Is the whole 2 minutes is still a thing if they don’t have all their teeth?

Parent 7

##### Design Suggestions

Parents suggested incorporating notifications, videos, engaging games, stories, and brushing timers into the app. They emphasized the need for brushing timers to be in the form of a song as children may not have a proper perception of time:

Kids don’t actually have the ability to understand time...I would say five more minutes till we got to leave the park. That doesn’t actually necessarily mean anything to a child, but following a song they would be able to know that they’re in the middle of the song.

Parent 6

Parents stressed the importance of using concise and straightforward language in the app and requested a simple design with the ability to return to the main screen at any time. They also suggested categorizing the app according to age for more targeted guidance. Incentives to download the app were discussed, with recommendations from health care professionals and trusted individuals being the most popular suggestions:

The midwifes, they give you like a list of some good apps on new-born babies or these are good apps for tracking how often they’re feeding.

Parent 2

I go to mother and baby groups, and they always have like posters or information. I guess if I saw something in that environment, I’d kind of be like, Oh, okay, maybe this is something that I should look at.

Parent 3

### Phase 3: Design and Develop a Prototype

**Figure 3:**
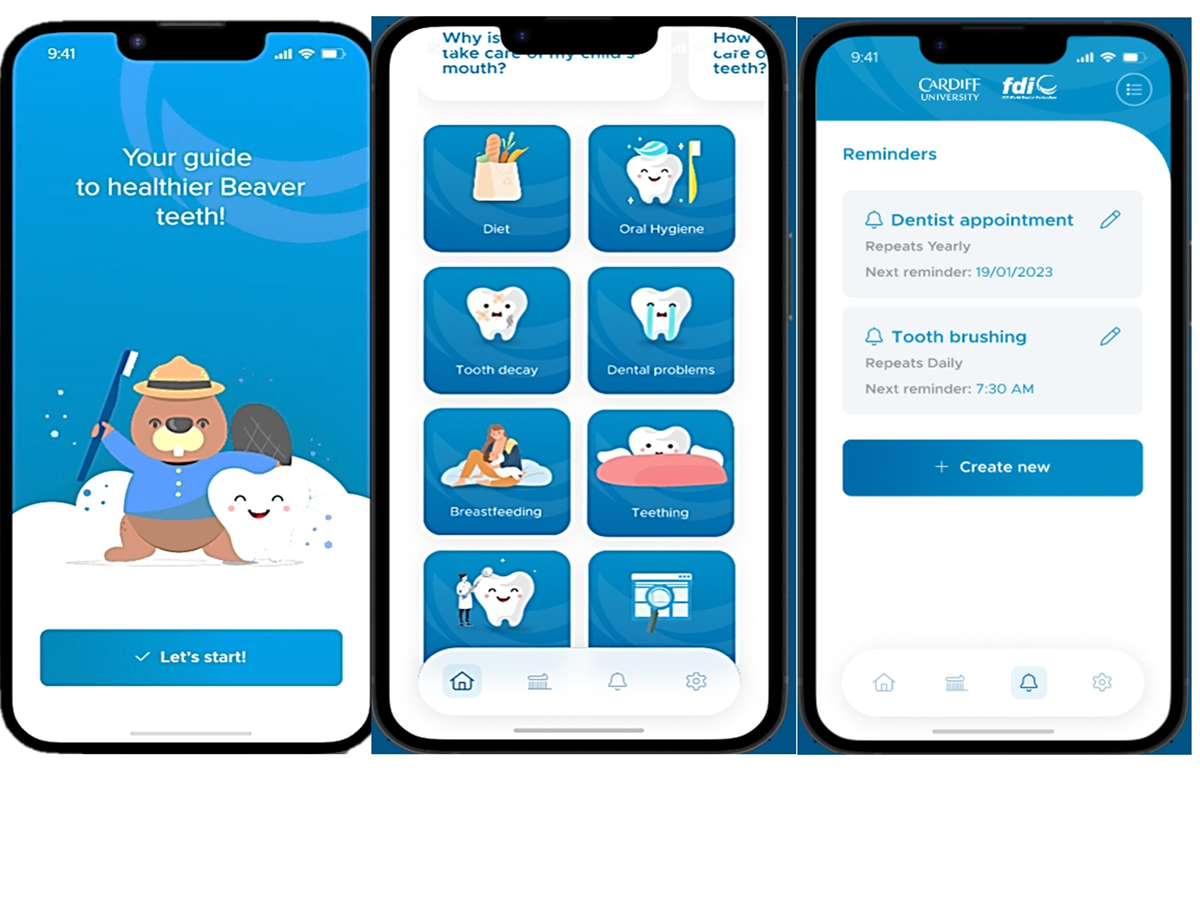
Overview of the App for Children’s Teeth interface.

The design stage of the study resulted in a prototype of an app named *App for Children’s Teeth* (ACT) based on parents’ views and their suggestions for content and design (Figure 3) The app was aligned with the Health Belief Model [14], including features to increase parents’ knowledge and self-efficacy regarding their children’s oral health practices as follows:

1. *Perceived susceptibility*—informs parents about the risks associated with poor oral health practices for their child’s overall health
2. *Perceived severity*—highlights the consequences of dental diseases if left untreated
3. *Perceived benefits*—educates parents about the benefits of proper oral health practices and incentivizes children with the stamps feature to brush their teeth regularly for better oral health outcomes
4. *Perceived barriers*—addresses common barriers to proper oral health practices, such as lack of knowledge, time, and resources, and provides strategies to overcome them, such as finding a local dentist
5. *Cues to action*—includes reminders and notifications to help parents establish good oral hygiene habits for their child
6. *Self-efficacy*—provides guidance and resources to help parents feel confident in their ability to perform proper oral health practices and prevent dental diseases from developing

### Phase 4: Assess the App’s Acceptability

#### Demographics

All participants, except for a woman parent (9/10, 90%), were interviewed for feedback about the app. Demographic information was similar to that in stage 1.

#### Main Findings

These data were gathered using the think aloud methodology by asking parents to share their thoughts about the design app prototype as they used it.

#### Content

The subjects that parents particularly wanted to see incorporated into the app included information about teething, how to brush children’s teeth, how much toothpaste to use, and what kind of toothpaste and toothbrush should be used. They also wanted a feature included to help them find a dentist. Although it was not possible to include everything in the prototype, they recognized when something they had asked about appeared in the app:

When I had my daughter, one of the things that I have researched the most was teething symptoms, signs, everything like that. The fact that it’s there and signs of it, how to alleviate. Amazing, love that.

Parent 3

#### Design

Parents felt that the structure fitted with their suggestions and it was simple, easy to navigate, and user-friendly. Having a unique character (the beaver) was perceived as a good way to engage the children with the app activities. A parent suggested allowing the child to name their beavers to enhance familiarity and, hence, engagement. Stamps provided upon completing brushing was also another feature that they felt would improve children’s engagement.

Other positive feedback about the design was having a “home button” on the screen all the time, which allows the users to go back to the main screen regardless of wherever they are. The font used (Sans Serif font) was found to be clear; however, a parent suggested checking the accessibility of the text and whether it is sufficiently clear for people with neurodiverse needs.

During the feedback interview, parents found several aspects of the app design to be useful, including the “In a Nutshell” bulletin points at the beginning of each subject, topics being categorized according to child’s age, and the ability to set reminders. In addition, some parents suggested sending notifications about new updates to keep parents engaged with the app for long term but with the ability for parents to control the frequency of these notifications:

You have reminders for toothbrushing. That’s really good for an older child you can get them to put their reminders on. I think they’ll like that. You could also have some notification, if new research comes out, for example, on a topic that you have in...not daily because people are likely to turn them off.

Parent 9

### TFA Questionnaire Analysis

As part of assessing the acceptability of the app, the TFA constructs were analyzed using the parent responses (Table 2).

**Table 2:** The Theoretical Framework of Acceptability (TFA) constructs, definitions, and their relevance to this study.

TFA constructs	Definition	Relevance to this study
Affective attitude	“Experienced Affective Attitude: How an individual feels about the intervention, after taking part”	Parents thought that the app prototype satisfied parents’ needs for oral health education, but because it is only a prototype, here is scope for improvement: “It’s very intuitive and I think parents will find this really help and easy to use. Obviously, there’s a few things to complete.” [Parent 9]
Ethicality	“The extent to which the intervention has good fit with an individual’s value system”	Parents found the app to be aligned with what they value about their children’s oral health: “I would say so, especially this day and age, everything is so busy. I rely on my phone for many things. It’s helpful to have an app that offers options for setting reminders and schedules.” [Parent 6] “The app is prioritising children, I think that’s brilliant.” [Parent 7]
Burden	“Experienced burden: the amount of effort that was required to participate in the intervention”	Thoughts about using an app such as ACT^a^ on a routine basis are variable; some parents thought that they can use such as app, but it will not be realistic to use it on a routine basis: “I don’t think a parent is likely to read about oral hygiene every day or every other day. You’ll read it if there’s an issue.” [Parent 5] Others felt that it provides convenience owing to its structure and being an app: “No not at all, I think something that you can download on your phone, you can choose a convenient time when you want to look at it and you can just access it any time and just look at the tabs that you want to, I think it’s really useful you’ve got all of the mini tabs, just to quickly click into, right at the front page, so you don’t even have to go digging for information because you can just choose what information you want to access straight away really, so yes I think it would be really useful.” [Parent 5]
Opportunity costs	“Experienced opportunity cost: the benefits, profits or values that were given up engaging in the intervention”	Participants feel that using such a tool will be beneficial to learn about oral health: “I think it will be easier because usually, whenever it’s a health-related issue about my children, I just Google things, but now I know there is one place for all information.” [Parent 1] It can also be a useful option if the dentist is not accessible: “If I couldn’t get on a list to go to a dentist which is a big problem for lots of people, it would maybe feel like, well, I can’t do that but at least- Here’s an app that’s giving me information that doesn’t feel like a stretch, I’ve downloaded this app so I’m doing something.” [Parent 9]
Perceived effectiveness	“Experienced effectiveness: the extent to which the intervention is perceived to have achieved its intended purpose”	ACT prototype was found to achieve its goals albeit having different priorities, but most parents emphasized the need to keep the app updated to continue achieving its objectives: “I’ve learned two things just by looking at it. but the information should be regularly updated, I suppose.” [Parent 7]
Self-efficacy	“The participant’s confidence that they can perform the behaviour(s) required to participate in the intervention”	Parents have different views about whether they feel confident that using the ACT app will keep them informed. Most cautious opinions where around whether the app follows the child’s growth: “You’d have to see more. it certainly will keep me informed because I have a resource to go to, but it would depend on the full functionality. As the child ages up, does the information change to stay current? Does it prompt you that maybe you should be buying a different type of toothbrush at this age?” [Parent 3]
Intervention coherence	“The extent to which the participant understands the intervention and how it works”	Parents thought that using technology such as an interactive app to achieve engagement with the target population (parents and young children) is a suitable intervention to improve children’s oral health: “Thinking of my daughter, now, I know that she loves interacting with apps because they have iPads at school. They have Chromebooks. So, I think children over five, maybe, who are in school already, would love to have something for them in there, like the one here, like the brushing, the videos, the timers and music and all that.” [Parent 9]

^a^ACT: App for Children’s Teeth.

### Suggestions for Further Development

A wide range of suggestions was made for the app’s next stages of development, but one of the main suggestions was around finding a dentist:

If you could outline the process of applying for a dentist, and the approximate wait times and things like that? I know that a lot of parents won’t be thinking about it until they need to do it.

Parent 4

Other suggestions were to enhance the app by addressing teeth shedding time, creating forum spaces for parent communication, and integrating augmented reality for guided brushing.

## Discussion

### Principal Findings

This study found that the interviewed parents felt there was a need for a reliable tool that they had confidence in, to assist them in managing their children’s oral health. This resonates with the worldwide trend of people using health apps to improve public health. With >55,000 health apps available globally on Google Store [15], these tools have, in some cases, been carefully evaluated to see how well they help with things such as diet [16,17], physical activity [18], or managing blood sugar [19]. mHealth apps are the most popular downloads from app stores.

They felt this would be particularly useful for young children who experience multiple landmarks during dental development such as teething, breastfeeding, and starting to brush their teeth.

An mHealth app for oral health was considered by them to be a viable possibility in filling this gap and would be easily accessible and useful for parents. Although a loose topic guide was used for the interviews, the parents were given the opportunity to talk broadly about oral health information, its sources, and the potential use of apps. Unsurprisingly, engaging the target population helped us understand their needs and preferences, and the iterative nature of the design process emphasized the need to continuously refine the prototype based on feedback. This is important in the development of digital health interventions such as mobile apps, which require a user-friendly interface and a seamless user experience to be effective.

### Health Literacy and Participants’ Demographics

Health literacy is a relatively new concept. It is “linked to literacy and entails people’s knowledge, motivation and competencies to access, understand, appraise and apply health information in order to make judgments and take decisions in everyday life concerning health care, disease prevention and health promotion to maintain or improve quality of life during the life course” [20]. It is known to be linked to better health outcomes, as individuals with high levels of health literacy are more able to navigate health care systems, advocate for themselves, and make good health and prevention-based choices [20]. Approximately half of the adults in the United States have been found to have a low or marginal level of health literacy [21], with similar findings in Europe [22]. With both the risk of dental caries and low health literacy being linked to socioeconomically disadvantaged groups, there is a risk that those who are most in need of easily available evidence-based preventive advice and oral health information are the least likely to be able to find, access, and use it. Using eHLS, the population that volunteered for this study showed an overall high level of health literacy and was likely not representative of the overall Welsh and UK populations. Nevertheless, they were in a good position to help inform the development of the prototype and comment about it. We tried to recruit different populations by accessing a group through university employees and a separate population in an area of Cardiff with low socioeconomic status. We used a community hub to invite participants and it may be that those who attend the hub are more likely to have reached a higher level of education and therefore health literacy than those who do not access the hub. For the further development stages of the app, we would actively seek a more representative group, possibly by accessing patient groups.

It was interesting that some of the parents mentioned screen time for their children as perhaps being a barrier to having the child engage with the app. The 2022 Ofcom Report about media use for parents and children found that in the UK nations, parents in Wales were more likely to be very concerned about their child’s media use [7,23].

### Design Process

#### Parents’ Current Source of Information

During the design interviews, parents reported that they primarily relied on search engines and government websites, such as the NHS website, for information about children’s oral health. However, they did not mention any specific resources dedicated to children’s oral health, indicating a need for better education and awareness about reliable sources of information. This may suggest that existing reliable resources have limited impact and reach, despite the funding and man power that are potentially invested in developing them. It will be crucial to develop a clear marketing plan during the further development stage of the app to increase its visibility. For example, some parents mentioned using apps related to pregnancy and parenting that were recommended by their midwives. This suggests that leveraging home visitors’ accessibility or dentists and general medical practitioners could be effective routes to promote the app. Parents also emphasized the value of peer experiences and word-of-mouth recommendations, as Facebook groups were frequently mentioned as a source of information and advice. In a similar way to mental health apps, leveraging social media platforms through health care providers might be a way to reach out to parents and promote the app through these channels [24]. Acknowledging the significance of trusted endorsements in mHealth apps for parents is crucial. In our study, all parents (10/10, 100%) consistently emphasized the importance of NHS endorsement as a key factor in their decision to download and trust the app. NHS holds weight and credibility as it signifies alignment with recognized health care standards. Therefore, it is essential to include a plan for obtaining such an endorsement during the design and development process. However, this journey is complex, lengthy, and multidisciplinary, involving registration as a medical device and adherence to NHS values and specifications.

#### Parents’ General Thoughts About the Development of an mHealth App for the Oral Health of Children

The parents were positive about the idea of an app for delivering oral health education to children. This positive reception was further evident in the recruitment process, which surpassed expectations, with parents actively providing feedback and suggestions. In addition, their willingness to receive follow-up emails showed their engagement. The enthusiastic response from parents highlights the potential impact of a well-designed and user-centric mHealth app in promoting positive health outcomes for children. By actively involving parents in the development and implementation processes, their perspectives and insights can be integrated into the app, ensuring better alignment with their expectations and health care needs.

#### Previous Experiences With Health Apps

Parents’ experiences with mHealth apps such as Headspace and MyFitnessPal provide valuable insights into the factors that contribute to their positive perceptions. The simplicity of these apps, characterized by user-friendly interfaces and intuitive navigation, resonated with parents seeking a hassle-free and user-friendly experience. The availability of free content in Headspace allowed parents to explore meditation and mindfulness practices without financial constraints, which was highly appreciated. Notifications related to mental health and well-being served as gentle reminders and motivators for parents to engage with the app. The wide applicability and provision of relevant information in MyFitnessPal were also highly valued by parents, highlighting the importance of addressing users’ needs and expectations. These insights were carefully considered and incorporated into the design stage of our app prototype, as demonstrated in the *Results* section.

### Development With an App Development Team

During the development of the dental app, collaboration with the app development team brought both excitement and the natural occurrence of differing priorities. Cross-disciplinary synergy was a highlight, with the app developers effectively responding to parent feedback by creating a user-friendly interface with quick 1-click buttons and a home button for easy navigation. However, differing priorities emerged with the app developers aiming for minimization of the number of screens for cost control, whereas the dental team prioritized clinical relevance and usability. These variations in focus were resolved through dialogue and compromise, leading to a better product.

### Feedback About the Prototype

The ACT app design and its content, created based on parents’ feedback and underpinned by the Health Belief Model, were felt by parents to have the potential to promote good oral health practices among children and parents. The positive findings of our project are consistent with the available evidence regarding the end user’s accessibility of using such technologies for oral health promotion [25-27]. Parents emphasized the importance of simplicity and user-friendliness of the app. These features were considered just as important as the quality of information provided in the app, highlighting the need to consider user experience in the design process and the importance of involving users in the development of health interventions. These findings are consistent with those of previous studies.

Key features of the app that were positively received included the incorporation of unique features such as a relatable character (the beaver). The character may have provided a sense of personalization and connection to the app. The “stamps” feature was designed based on gamification principles, providing a tangible and visible reward for completing oral-related tasks [9]. This type of engagement strategy has been shown in a meta-analysis to be effective in promoting positive oral health behavior change, such as regular toothbrushing and flossing, among children and parents [28]. The reminder and notification feature of the ACT app was positively received by parents, indicating its potential value in promoting long-term engagement with the app. Reminders and notifications can increase engagement and retention in digital health interventions and can lead to improved health outcomes.

Although the app has several positive features, some feedback highlighted the aspects to be considered for future development. Many parents (6/10, 60%) expressed the need for NHS endorsement as a crucial factor in engaging with the app. Securing NHS endorsement poses significant challenges owing to the stringent standards and guidelines established by NHS [29]. These complexities span clinical validation, regulatory adherence, incorporation of user feedback, engagement with stakeholders, conducting health economic assessments, and intricate management of legal and ethical considerations. Each of these facets demand meticulous attention and allocation of resources to align with NHS’s rigorous criteria and secure their indispensable support. Therefore, careful planning and consideration of these factors are necessary in further development stages.

A parent also commented about the accessibility considerations of the app for individuals with neurodiverse needs. Incorporating accessible, evidence-based recommendations into the design process can ensure that the app reaches a broad audience and promotes better oral health outcomes [25].

Finally, concerns about screen time were raised by parents. Studies have highlighted the adverse impacts of excessive screen time on various aspects of children’s well-being, including cognitive and socioemotional development [30,31].

Hence, to ensure successful adoption and sustained use of the mHealth app, it is essential to address these concerns and mitigate the potential impact of increased screen time. Strategies can be implemented within the app to promote responsible screen time practices. This may include incorporating features that allow parents to set time limits on app use, providing guidance about optimal screen time duration for different age groups, and emphasizing the importance of engaging in offline activities and interactions to complement the app’s use [32]. Including ideas for interactive, off-screen activities such as games and quizzes may also be beneficial [33].

### Limitations

The study’s results may have been limited by several factors. First, most of the participants (8/10, 80%) were highly educated women, most (participants had university education; 9/10, 90%), and the age range was relatively narrow, with all participants (10/10, 100%) aged between 25 and 44 years. This was probably related to the recruitment strategy despite efforts to recruit diverse groups by targeting both a university employee cohort and local community center members.

Furthermore, the recruitment process relied on convenience sampling, which may have resulted in a sample that is not representative of the broad population. Although the researchers made efforts to recruit participants through multiple channels, all participants (10/10, 100%) were from 1 city, which may limit the applicability of the findings. This is particularly relevant because the app was developed in collaboration with the International Dental Federation (Fédération Dentaire Internationale), which aims to further refine the app with global perspective.

It is crucial to highlight that the exclusion of children as participants in our study significantly hampers our comprehension of their perspectives regarding the app. This is particularly important considering that parents expressed the belief that the app should be used collaboratively by both the parent and the child. By omitting children from the study, we miss valuable insights that could contribute to a more comprehensive understanding of the app’s use dynamics.

In addition, the development process may have been constrained by limited funding and man power, which may have influenced the extent of user testing and refinement of the app. However, despite these limitations, the study provides valuable insights into the importance of user-centered design and testing in the development of mHealth apps. Future studies can use these insights to further refine and evaluate the effectiveness of the app in improving oral health outcomes in diverse populations.

### Conclusions

Digital health interventions, such as the ACT app, have the potential to promote healthy behaviors among children and have important implications for public health. The incorporation of theoretical framework, user cocreation in the design process, and emphasis on simplicity and user-friendliness have been identified as key factors contributing to the success of the intervention. These strategies have also been shown to be effective in promoting engagement and adoption of the intervention. Further studies are needed to build the app based on parents’ views and pilot test to evaluate its impact on promoting healthy behaviors among children.

The study highlights the importance of user-centered design when developing health-related tools and the value of conducting user research and iterative testing to ensure that the tool meets the needs of the target audience. The findings of this study can inform the development of similar tools in the future and ultimately help to improve oral health outcomes for young children.

## Appendix

Multimedia Appendix 1 Document to engage participants.

Multimedia Appendix 2 Registration form.

Multimedia Appendix 3 Participant information sheet.

Multimedia Appendix 4 Consent form.

Multimedia Appendix 5 Interview guide.

Multimedia Appendix 6 Example of the mobile app’s layout.

Multimedia Appendix 7 Participants’ responses to the eHealth Literacy Scale (eHLS) questions (Qs; A) 1 and (B) 2 (n=9).

## Abbreviations

ACT: App for Children’s Teeth
eHLS: eHealth Literacy Scale
mHealth: mobile health
NHS: National Health Service
TFA: Theoretical Framework of Acceptability

## Data Availability

Preliminary data are available upon reasonable requests.
