# Supplementary material for: “I Just Wanted a Dentist in My Phone”—Designing Evidence-Based mHealth Prototype to Improve Preschool Children’s Oral and Dental Health: Multimethod Study of the Codevelopment of an App for Children’s Teeth
Source: JMIR Form Res. 2024 Jan 30;8:e49561. doi: 10.2196/49561 (PMC10865186; doi:10.2196/49561)

**
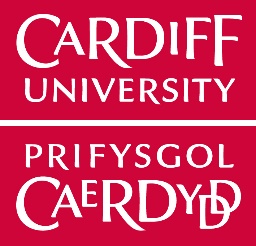
Document to engage participants**

**“An app for children’s teeth - ACT”**

**(Webpage – Cardiff University Yammer and other media, Grangetown Community Centre)**

If you are the parent or carer of a child who is 6 years old or younger, we need your help with our study! We are making a mobile phone app to help parents take care of their young children’s teeth. We want to find out what they think would be useful and ask them to try out our new app.

**"**

**"**

Tooth decay and other dental issues are a big problem for children. Many can be prevented or picked up early when parents and carers know what to do and look for in children’s mouths. To help with this, we are developing a mobile phone App focusing on children’s oral health to help parents and carers keep children’s mouths healthy and prevent tooth decay. One of the most important things for online information resources and health-related Apps to be successful is a good end-user experience. We want to develop the App and check its acceptability with the help of parents of children 6 years old and under.

To take part in this study we will need you to have two meetings with the researchers for a recorded interview. These will take place between September and November. Each one will last less than an hour. The researcher will ask you about caring for your children’s teeth and what information would help you. They will also show you the App and how it works, and they will ask you to make suggestions and give your thoughts on it.

This research is funded by “Innovation for All”, Cardiff University. The Dental School Research Ethics Committee have given the study a favourable ethics review.

We would be very grateful for your help in carrying out this research. Your participation is entirely voluntary, you may choose to withdraw your participation at any time.

If you would like to take part in the research, please click on the link to register your interest and check if you are eligible to participate. We will contact you shortly afterwards if you are eligible on the registration form.

If you would like more details, please contact Daniela Raggio: raggiod@cardiff.ac.uk

Thank you!

Dr Daniela Raggio (Senior Lecturer, School of Dentistry), Prof Nicola Innes (Honorary Consultant in Paediatric Dentistry and Head of School of Dentistry) and Dr Waraf Al-Yaseen (Clinical Lecturer, School of Dentistry)

<https://forms.office.com/Pages/ResponsePage.aspx?id=MEu3vWiVVki9vwZ1l3j8vOf_IHtr6BdLjQOTouCJs55UMktLS0MxREdZNjBXSzNFSzNSUElDTlYzWi4u>


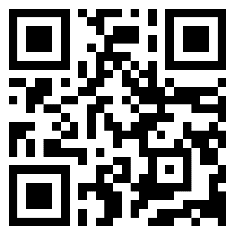

Supplement: Multimedia Appendix 1 [file formative_v8i1e49561_app1.docx]
