# Supplementary figures and images for: “I Just Wanted a Dentist in My Phone”—Designing Evidence-Based mHealth Prototype to Improve Preschool Children’s Oral and Dental Health: Multimethod Study of the Codevelopment of an App for Children’s Teeth
Source: JMIR Form Res. 2024 Jan 30;8:e49561. doi: 10.2196/49561 (PMC10865186; doi:10.2196/49561)

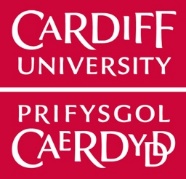
 **Appendix 2 – Registration form**

**“An app for children’s teeth - ACT”**


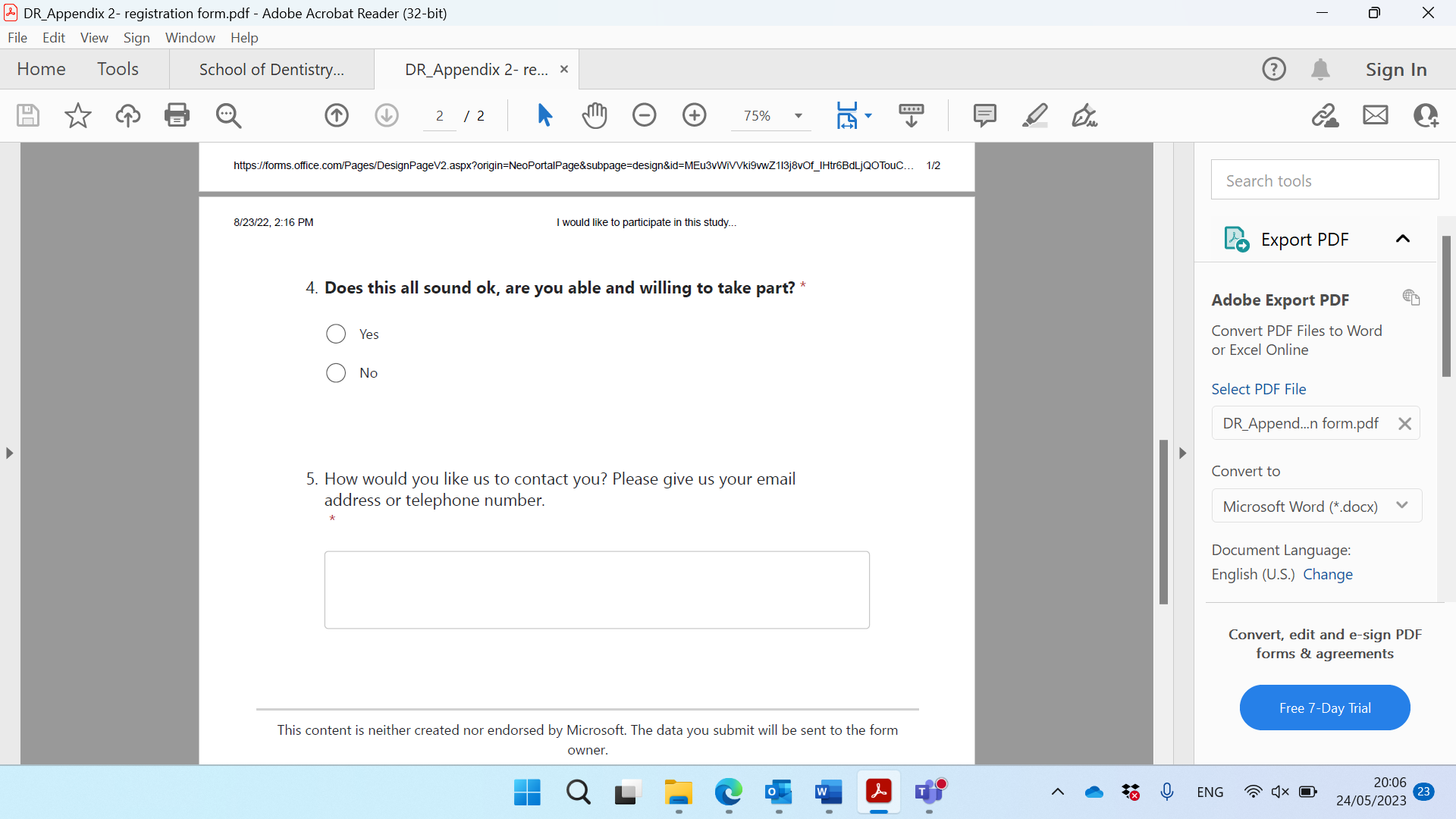

Supplement: Multimedia Appendix 2 [file formative_v8i1e49561_app2.docx]

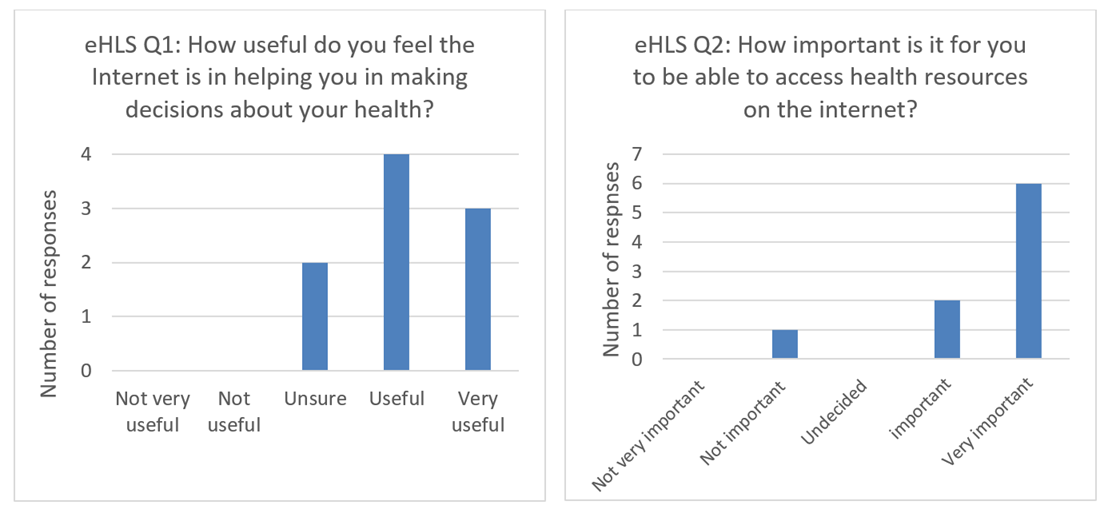

Supplement: Multimedia Appendix 7 [file formative_v8i1e49561_app7.png]
