# Supplementary material for: “I Just Wanted a Dentist in My Phone”—Designing Evidence-Based mHealth Prototype to Improve Preschool Children’s Oral and Dental Health: Multimethod Study of the Codevelopment of an App for Children’s Teeth
Source: JMIR Form Res. 2024 Jan 30;8:e49561. doi: 10.2196/49561 (PMC10865186; doi:10.2196/49561)
Supplement: Multimedia Appendix 3 [file formative_v8i1e49561_app3.docx]

**Appendix 3**
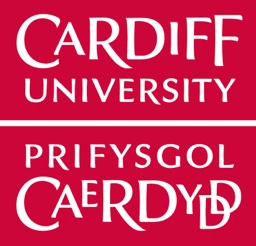
 **PARTICIPANT INFORMATION SHEET**

**“An app for children’s teeth - ACT”**

You are being invited to take part in a research project. Before you decide whether to take part, it is important for you to understand why the research is being undertaken and what it will involve. Please take time to read the following information carefully and discuss it with others if you wish.

Thank you for reading this.

1. **What is the purpose of this research project?**

This research project is developing an “App” for parents and carers of young children (6 years old and younger). It will provide information on keeping their child’s mouth healthy and how to find (and try to solve) any problems early. We need help from people who might use the App to check if the information is easy to understand and presented well. We also need to know whether it is useful and how to improve it.

We will do this by having an initial interview meeting (less than 60 minutes) with parents/carers. At this meeting we will ask about how you find information on children’s oral and tooth health. We will also ask about what kind of mobile devices you use to get information and your experience of using apps for health purposes. We will ask you for ideas about the design of the app. You will need to answer a demographic questionnaire and your thoughts will be audio recorded. Using your feedback, we will develop and adapt the first version of the App.

We will arrange a second visit (maximum 60 minutes) when you will have the chance to try out the App for 10 minutes, and then ask you more questions, focusing on what you think of the App’s appearance, content, and language appropriateness.

1. **Why have I been invited to take part?**

You have been invited because you are a parent or carer of a child who is 6 years-old (or younger) and you completed a form online to tell us you were interested in taking part in our study.

1. **Do I have to take part?**

No, your participation in this research project is entirely voluntary and it is up to you to decide whether to take part. If you decide to take part, we will discuss the research project with you and ask you to sign a consent form. If you decide not to take part, you do not have to explain your reasons and it will not affect your legal rights.

You are free to withdraw your consent to participate in the research project at any time, without giving a reason, even after signing the consent form.

1. **What will taking part involve?**

You will need to attend two meetings in person. They will last less than 60 minutes each, and there is no need for your child to attend the meetings.

1. **Will I be paid for taking part?**

Yes, as a thank you for your time and help, you will receive a £30 voucher for each meeting. This will be £60 in total for both meetings. You should understand that any data you give will be as a gift and you will not benefit financially in the future should this research project lead to the development of anything new.

1. **What are the possible benefits of taking part?**

You might find out information that might help your child’s oral health or teeth and from talking with a children’s dentist about children’s teeth. Your contribution to the project will help us to improve the App content and layout by understanding how parents of young children feel about the oral health application, and how it can be used better to build more healthy behaviours. The App will help other people look after children’s teeth better.

1. **What are the possible risks of taking part?**

There are no foreseeable discomforts, risks, or disadvantages of taking part in this research apart from the time it will take for the meetings and trying out the App with the researcher.

1. **Will my taking part in this research project be kept confidential?**

All information collected from you during the research project will be kept confidential and any personal information you provide will be managed in accordance with data protection legislation. Please see ‘What will happen to my Personal Data?’ (below) for further information.

1. **What will happen to my Personal Data?**

Study data will be analysed via a main data file that will be held securely; your name or any contact details will not be included in this file, just a subject identification number will be allocated.

Cardiff University is the Data Controller and is committed to respecting and protecting your personal data in accordance with your expectations and Data Protection legislation. Further information about Data Protection, including:

- your rights
- the legal basis under which Cardiff University processes your personal data for research
- Cardiff University’s Data Protection Policy
- how to contact the Cardiff University Data Protection Officer
- how to contact the Information Commissioner’s Office

may be found at https://www.cardiff.ac.uk/public-information/policies-and-procedures/data-protection

After completing the interviews, the research team will anonymise all the personal data it has collected from, or about, you in connection with this research project, except for your consent form. Your consent form will be retained for 5 years and may be accessed by members of the research team and, where necessary, by members of the University’s governance and audit teams or by regulatory authorities.   Anonymised information will be kept in accordance with the University Records Retention Schedules but may be published in support of the research project and/or retained indefinitely, where it is likely to have continuing value for research purposes.

You can withdraw from the research at any time. Note that it will not be possible to withdraw any anonymised data that has already been published or in some cases, where identifiers are irreversibly removed during a research project, from the point at which it has been anonymised.

1. **What happens to the data at the end of the research project?**

We will follow the GDPR guidelines for data protection (2008) to handle and store data. Transcripts will be stored in a password protected University cloud space and will be accessible only for the research team. After 5 years, data will be destroyed according to the University procedures.

1. **What will happen to the results of the research project?**

It is our intention to publish the results of this research project in academic journals and present findings at academic conferences. Participants will not be identified in any report, publication, or presentation. Nevertheless, there is an intention to use verbatim quotes from participants.

1. **What if there is a problem?**

If you wish to complain or have grounds for concerns about any aspect of the way you have been approached or treated during this research, please contact Daniela Procida Raggio (raggiod@cardiff.ac.uk). If your complaint is not managed to your satisfaction, please contact the Chair of the Dental School Research Ethics Committee (Dr Damian Farnell: FarnellD@cardiff.ac.uk).

If you are harmed by taking part in this research project, there are no special compensation arrangements. If you are harmed due to someone's negligence, you may have grounds for legal action, but you may have to pay for it.

1. **Who is organising and funding this research project?**

The research is organised by a research team, led by Dr Daniela Procida Raggio, School of Dentistry in Cardiff University. Prof Nicola Innes and Dr Waraf Al-Yaseen are the other researchers. The research is currently funded by Innovation for All 2022 Scheme, Cardiff University.

1. **Who has reviewed this research project?**

This research project has been reviewed and given a favourable opinion by the Dental School Research Ethics Committee.

1. **Further information and contact details**

Should you have any questions relating to this research project, you may contact us during normal working hours:

Daniela Procida Raggio, Senior Lecturer on Clinical Dental Research, School of Dentistry, Cardiff University. University Dental Hospital (UDH), Heath Park, Cardiff. CF14 4XW

Email: [raggiod@cardiff.ac.uk](mailto:raggiod@cardiff.ac.uk)

Phone number: +44 7423 460126

**Thank you for considering taking part in this research project. If you decide to participate, you will be given a copy of the Participant Information Sheet and a signed consent form to keep for your records.**
