# Supplementary material for: “I Just Wanted a Dentist in My Phone”—Designing Evidence-Based mHealth Prototype to Improve Preschool Children’s Oral and Dental Health: Multimethod Study of the Codevelopment of an App for Children’s Teeth
Source: JMIR Form Res. 2024 Jan 30;8:e49561. doi: 10.2196/49561 (PMC10865186; doi:10.2196/49561)
Supplement: Multimedia Appendix 4 [file formative_v8i1e49561_app4.docx]

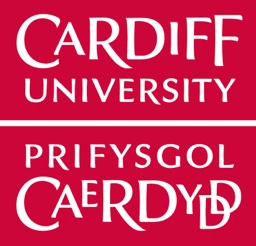
**Appendix 4. Consent Form**

**Title of research project: Development of an Evidence-Based Mobile Health Application for, and with, Parents to Improve Children’s Oral Health**

**Short title: An app for children’s teeth - ACT**

DSREC reference and committee

Name of Chief/Principal Investigator: Daniela Raggio

|  | **Please initial box** |
| --- | --- |
| I confirm that I have read the information sheet dated 29^th^ September 2022 for the above research project. |  |
| I confirm that I have understood the information sheet dated 29^th^ September 2022 for the above research project and that I have had the opportunity to ask questions and that these have been answered satisfactorily. |  |
| I understand that my participation is voluntary, and I am free to withdraw at any time without giving a reason and without any adverse consequences (e.g., to medical care or legal rights, if relevant). I understand that if I withdraw, information about me that has already been obtained may be kept by Cardiff University. |  |
| I understand who will have access to personal information provided, how the data will be stored and what will happen to the data at the end of the research project. |  |
| I understand that after the research project, anonymised data may be made publicly available via a data repository and may be used for purposes not related to this research project. I understand that it will not be possible to identify me from this data that is seen and used by other researchers, for ethically approved research projects, on the understanding that confidentiality will be maintained. |  |
| I consent to being audio recorded for the purposes of the research project and I understand how it will be used in the research. |  |
| I understand how the findings and results of the research project will be written up and published. |  |
| I agree to take part in this research project. |  |

Name of participant (print) Date Signature

Name of person taking consent Date Signature

(print)

**_________________________**

**Role of person taking consent**

**(print)**

**THANK YOU FOR PARTICIPATING IN OUR RESEARCH**
