# Supplementary material for: “I Just Wanted a Dentist in My Phone”—Designing Evidence-Based mHealth Prototype to Improve Preschool Children’s Oral and Dental Health: Multimethod Study of the Codevelopment of an App for Children’s Teeth
Source: JMIR Form Res. 2024 Jan 30;8:e49561. doi: 10.2196/49561 (PMC10865186; doi:10.2196/49561)
Supplement: Multimedia Appendix 5 [file formative_v8i1e49561_app5.docx]

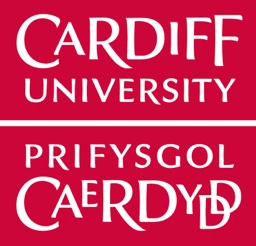
**Appendix 5**

**“An app for children’s teeth - ACT”**

1. What gender do you identify as?
2. Please describe your race/ethnicity.
3. What is your age range?

- 18-24
- 25-34
- 35-44
- 45-54
- Above 55

1. What is the highest level of education you have completed?

- Did not attend school
- Attended high school
- Attended college course
- Attended university course
- Other post-graduate education

**Topic: Digital literacy - eHealth Literacy Scale** (Norman and Skinner, 2006)

I would like to ask you for your opinion and about your experience using the Internet for health information. For each statement, tell me which response best reflects your opinion and experience *right now*.

**1. How useful do you feel the Internet is in helping you in making decisions about your health?**

| o1 | o2 | o3 | o4 | o5 |
| --- | --- | --- | --- | --- |
| Not useful at all | Not useful | Unsure | Useful | Very Useful |

**2. How important is it for you to be able to access health resources on the Internet?**

| o1 | o2 | o3 | o4 | o5 |
| --- | --- | --- | --- | --- |
| Not important at all | Not important | Unsure | Important | Very important |

**3. I know what health resources are available on the Internet**

o Strongly Disagree o Disagree o Undecided o Agree o Strongly Agree

**4. I know where to find helpful health resources on the Internet**

o Strongly Disagree o Disagree o Undecided o Agree o Strongly Agree

**5. I know how to find helpful health resources on the Internet**

o Strongly Disagree o Disagree o Undecided o Agree o Strongly Agree

**6. I know how to use the Internet to answer my questions about health**

o Strongly Disagree o Disagree o Undecided o Agree o Strongly Agree

**7. I know how to use the health information I find on the Internet to help me**

o Strongly Disagree o Disagree o Undecided o Agree o Strongly Agree

**8. I have the skills I need to evaluate the health resources I find on the Internet**

o Strongly Disagree o Disagree o Undecided o Agree o Strongly Agree

**9. I can tell high quality health resources from low quality health resources on the Internet**

o Strongly Disagree o Disagree o Undecided o Agree o Strongly Agree

**10. I feel confident in using information from the Internet to make health decisions**

o Strongly Disagree o Disagree o Undecided o Agree o Strongly Agree
