# Supplementary material for: “I Just Wanted a Dentist in My Phone”—Designing Evidence-Based mHealth Prototype to Improve Preschool Children’s Oral and Dental Health: Multimethod Study of the Codevelopment of an App for Children’s Teeth
Source: JMIR Form Res. 2024 Jan 30;8:e49561. doi: 10.2196/49561 (PMC10865186; doi:10.2196/49561)
Supplement: Multimedia Appendix 6 [file formative_v8i1e49561_app6.docx]

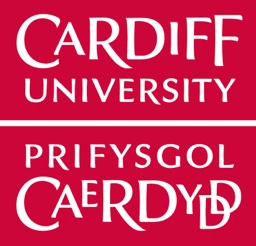
 **Appendix 6: Example of the mobile application layout**

**“An app for children’s teeth - ACT”**


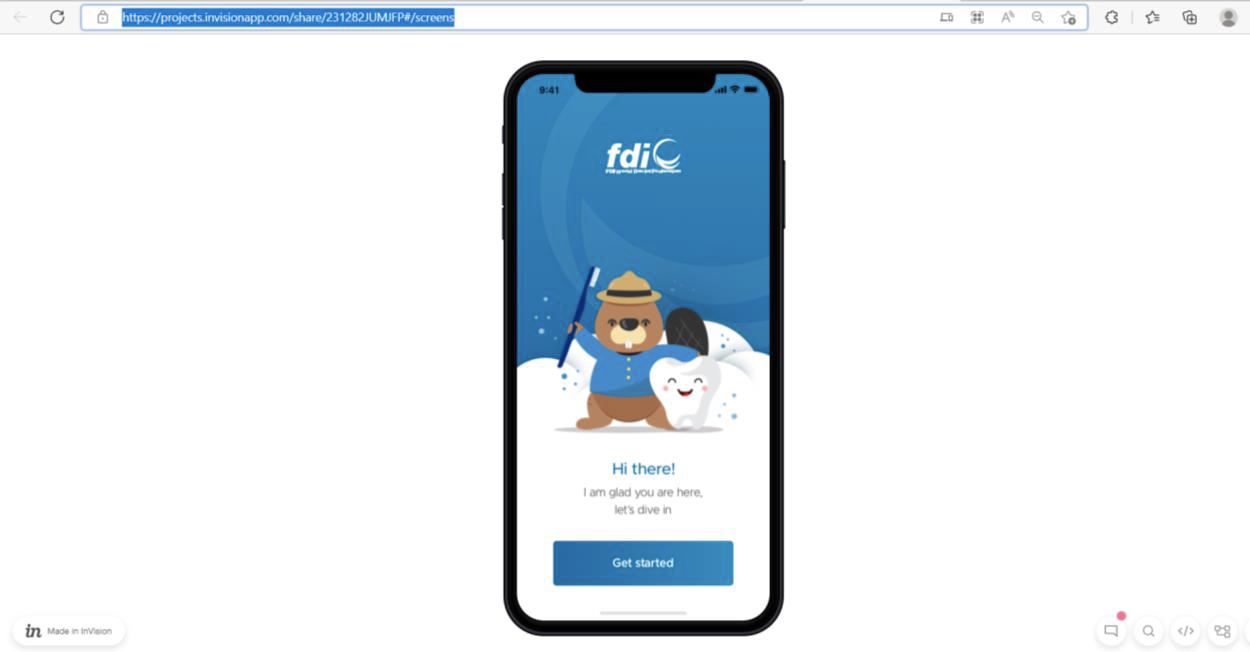


*Screen 1*


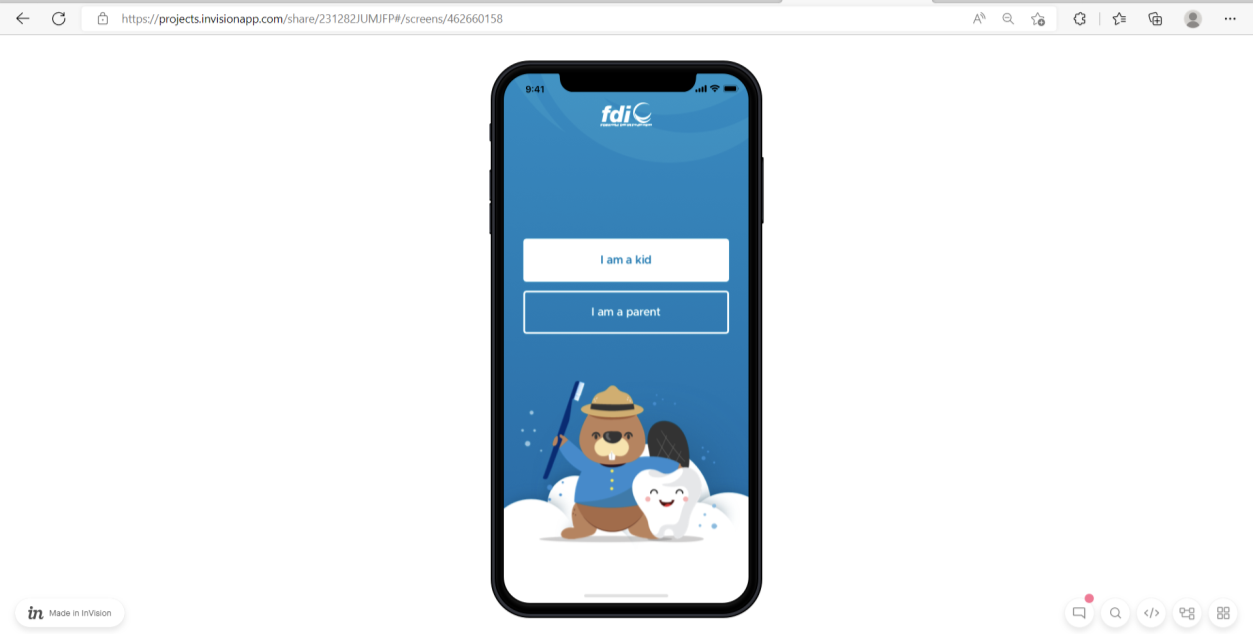


Screen 2.


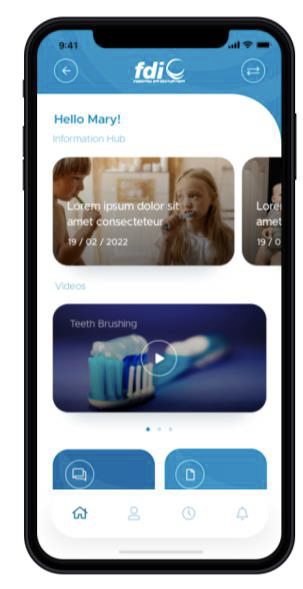

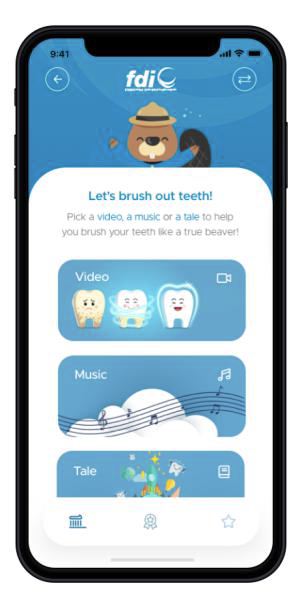

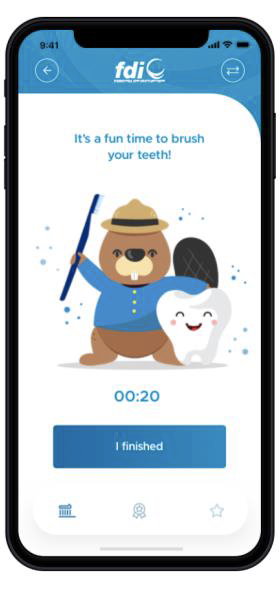

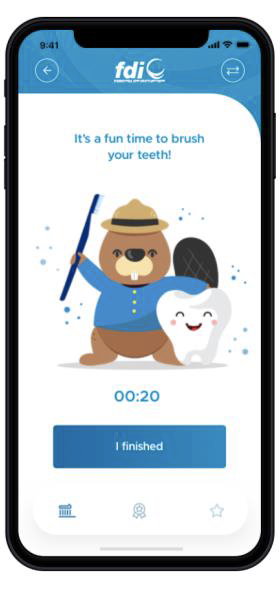


3 parent screen 4 Child screen 5 Child screen 6 Child screen
